# Reconstruction of Critical Sized Maxillofacial Defects Using Composite Allogeneic Tissue Engineering: Systematic Review of Current Literature

**DOI:** 10.3390/biomimetics8020142

**Published:** 2023-03-30

**Authors:** Shaqayeq Ramezanzade, Mahsa Aeinehvand, Heliya Ziaei, Zohaib Khurshid, Seied Omid Keyhan, Hamid R. Fallahi, James C. Melville, Morvarid Saeinasab, Farshid Sefat

**Affiliations:** 1 Section for Clinical Oral Microbiology, Department of Odontology Cariology and Endodontics, University of Copenhagen, DK-2200 Copenhagen, Denmark; 2 Maxillofacial Surgery & Implantology & Biomaterial Research Foundation, Tehran P.O. Box 14155-6559, Iran; 3 Department of Prosthodontics and Dental Implantology, King Faisal University, Al-Ahsa 31982, Saudi Arabia; 4 Center of Excellence for Regenerative Dentistry, Department of Anatomy, Faculty of Dentistry, Chulalongkorn University, Bangkok 10330, Thailand; 5 Maxillofacial Surgery & Implantology & Biomaterial Research Foundation, Isfahan P.O. Box 61355-45, Iran; 6 Oral, Head & Neck Oncology and Microvascular Reconstructive Surgery, Department of Oral and Maxillofacial Surgery, School of Dentistry, University of Texas Health Science Center at Houston, Houston, TX 77030, USA; 7 Department of Biomedical and Electronic Engineering, School of Engineering, University of Bradford, Bradford BD7 1DP, UK

**Keywords:** tissue engineering, maxillofacial defects, composite graft

## Abstract

The current review aimed to assess the reliability and efficacy of tissue-engineered composite grafts in the reconstruction of large maxillofacial defects resulting from trauma or a benign pathologic disease. A systematic review of the literature was conducted using PubMed/Medline, Embase, and Scopus up to March 2022. The eligibility criteria included patients who had been treated with composite allogeneic tissue engineering for immediate/delayed reconstruction of large maxillofacial defects with minimum/no bone harvesting site. In the initial search, 2614 papers were obtained, and finally, 13 papers were eligible to be included in the current study. Most included papers were case reports or case series. A total of 144 cases were enrolled in this systematic review. The mean age of the patients was 43.34 (age range: 9–89). Most studies reported a successful outcome. Bone tissue engineering for the reconstruction and regeneration of crucial-sized maxillofacial defects is an evolving science still in its infancy. In conclusion, this review paper and the current literature demonstrate the potential for using large-scale transplantable, vascularized, and customizable bone with the aim of reconstructing the large maxillofacial bony defects in short-term follow-ups.

## 1. Introduction

The oral and maxillofacial area is a complex region including osseocartilaginous elements, neural and vascular systems, skin, and other lining and covering tissues, teeth, and organs for the senses [1]. There are several causes of significant defects in this region, such as traumatic avulsion, Osteoradionecrosis (ORN), bisphosphonate-related osteonecrosis of the jaws (BRONJ), the resection of benign/malignant tumors and cysts, etc. [2]. The natural repair mechanisms for large maxillofacial defects are insufficient and slow-paced [3]. Therefore, adjunct bone regeneration procedures are crucial to ensure sufficient bone formation within a short time.

Materials of natural origin, derived from a living source without making any modifications consist of four major groups: autografts, allografts, xenografts, and phytogenic materials [4]. The current routine materials of reconstruction include autologous cortico-cancellous bone, vascularized free flap transfer, alloplastic materials with prosthetic appliances, and composite materials [5]. 

The techniques advocated for each case depend on the associated soft tissue, the pattern of vascularity, defect size, the types of tissue, and patient preference [6,7].

Reconstruction of large maxillofacial defects with conventional materials and techniques of autogenous bone collection presents a set of challenges for the surgeon in the maxillofacial field; the amount of intraoral bone is mostly limited and therefore is not suitable for harvesting and grafting large defects [4]. Likewise, the need for another surgical site results in burdensomely long and complex operations, hospital stay, higher rates of post-surgical complications, and the morbidities of the bone harvesting sites. 

A widely used alternative option for bone regeneration is the use of alloplastic materials, which eliminates the need for a donor site and improves surgical efficiency. This approach is also much safer in medically compromised patients, in which the risks of additional graft harvesting surgery outweigh the benefits. The macroporosity of 100 to 400 mm on the surface of such materials acts as trabecular bone and therefore promotes osteoconduction [8]. At the same time, the lack of cellular components required for osteogenesis and weak activity in vascularly compromised environments are counted as major flaws [1]. 

Mesenchymal stem cells (MSCs) derived from different parts of the human body such as bone marrow, adipose tissue, peripheral blood, etc., have shown an enhancement in bone regeneration when seeded on a scaffold compared to an unseeded scaffold alone [9].

Using an allogenic graft as a biologic scaffold in conjunction with harvested mesenchymal stem cells and recombinant human bone morphogenic protein-2 (rhBMP-2) creates a favorable microenvironment for bone formation. This review aimed to assess the reliability and efficacy of tissue-engineered composite grafts in the reconstruction of large maxillofacial defects of trauma or a benign pathologic disease.

## 2. Materials and Methods

### 2.1. Protocol Registration

The search protocol was specified and registered at PROSPERO (prospective international register of systematic reviews) with registration number: CRD42021242399. In addition, the PRISMA 2020 guidelines for conducting this systematic review were followed [10].

### 2.2. PICO Question

Patient: patients with large maxillofacial defects requiring bone regeneration.

Intervention: surgical bone grafting procedures using composite allogeneic tissue engineering.

Comparison: Conventional autogenous bone grafts/None (non-comparative studies).

### 2.3. Outcome

1. The complication rates reported.
2. The success rate measured as the amount of new bone volume gained (assessed either directly by percentage bone fill or assessed radiographically).
3. Patient-centered outcomes: satisfaction rate.

### 2.4. Search Strategy

PubMed/Medline, Embase, and Scopus were searched systematically with no time and language restrictions (up to March 2022) [2]. Also, the reference list of included papers was hand-searched for potential additional papers. Table 1 illustrates the search strategy for each database.

### 2.5. Inclusion Criteria

The inclusion criteria of the current review were as follows:

1. Original studies, written in English, including randomized controlled trials (RCTs), Clinical trials, observational studies (cohorts and case series) as well as case reports on human patients who had been treated with composite allogeneic tissue engineering for immediate/delayed reconstruction of large maxillofacial defects with minimum/no bone harvesting site.
2. The composite allogeneic tissue engineering was defined as a combination of allogenic bone (scaffolding), bone morphogenic aspirate (source of stem cells), rhBMP-2, and platelet-rich plasma/platelet-rich fibrin (cell signaling for the promotion of stem cell migration and differentiation into osteoblasts).
3. No minimum follow-up was established.
4. Studies must report on at least one of the outcomes of interest:
  - The complication rates were reported. Either early post-surgical complications or long-term post-surgical complications.
  - The success rate is measured as the amount of new bone volume gained (assessed either directly by gross observation or assessed radiographically).
  - Patient-centered outcomes: satisfaction rate and esthetic and functional results.

The Exclusion criteria were as follows (the reasons for excluding articles are also recorded in Table 2):

1. Nonhuman study and cadaver studies.
2. Studies involving significant autogenous bone grafts from sites like the ilium, rib, fibula, or calvarium.

### 2.6. Study Selection Process

In order to determine proper materials, two reviewers conducted a duplicate searching process using the inclusion and exclusion criteria independently. Instances of divergence of opinion were resolved by consulting a third investigator (Sh.R.). The full-text version of papers was obtained for all titles that appeared to meet the inclusion criteria or in case of any hesitancy. Then, each paper was studied at least twice by two reviewers (M.A. and H.Z.). 

### 2.7. Data Extraction

Whenever applicable, two authors (M.A. and H.Z.) retrieved the following data from the finally included studies based on a predefined paper checklist, and three other authors (Sh.R., Z.Kh., and J.M.) supervised the extraction process for accuracy. Since poorly reported outcomes of included materials could thread the validity of our work, we contacted the corresponding author of the study via email, sending up to two emails, in case of missing data or any hesitancy. The following data were extracted: 

First author, year of publication, country of origin, study type, mean age, sex, number of cases, mean follow-up (range), gained bone volume, rates of complications, donor-site morbidities, and success rates and main outcomes.

### 2.8. Risk of Bias Assessment

Two examiners (M.A. and H.Z.) conducted the quality assessment according to the following quality assessment tools and supervised by a third author (Sh.R.) for accuracy. Any disagreement was resolved by consensus (Table 3). 

The methodological quality and synthesis of case series and case reports by Murad et al. were used for bias assessment [11,12,13,14]. Summing the scores and presenting an aggregate score was not appropriate, and making an overall judgment about the quality should be based on the most critical questions.

**Table 3:** Risk of bias assessment for case series and case reports.

First Author (Year of Publication)	1. Does the Patient(s) Represent(s) the Whole Experience of the Investigator (Centre) or Is the Selection Method UNCLEAR to the Extent That Other Patients with Similar Presentation May Not Have Been Reported?	2. Was The Exposure Adequately Ascertained?	3. Was the Outcome Adequately Ascertained?	4. Were Other Alternative Causes that May Explain the Observation Ruled Out?	5. Was There a Challenge/Rechallenge Phenomenon?	6. Was There a Dose–Response Effect?	7. Was Follow-up Long Enough for Outcomes to Occur?	8. Is the Case(s) Described with Sufficient Details to Allow Other Investigators to Replicate the Research or to Allow Practitioners to Make Inferences Related to Their Own Practice?
James C. Melville, 2016, Houston, English [15]	N	Y	Y	N	Y	NA	Y	Y
James C. Melville, 2017, Houston, English [16]	N	Y	Y	N	Y	NA	Y	Y
J. C. Melville, 2014, Houston, English [17]	N	Y	Y	N	Y	NA	Y	Y
James C. Melville, 2019, Houston, English [18]	N	Y	Y	N	Y	NA	Y	Y
Matthias Schlund, 2019, Oman, English [19]	N	Y	Y	N	Y	NA	Y	N
Kamel Alraei, 2020, Saudi Arabia, English [20]	N	Y	Y	N	Y	NA	Y	Y
Jeanette Johnson, 2016, Texas, English [21]	N	Y	Y	N	Y	NA	Y	Y
N. Ali, 2018, Texas, English [22]	N	Y	Y	N	Y	NA	Y	Y
Todd G. Carter, 2008, Seattle, English [23]	N	Y	Y	Y	Y	NA	Y	Y
Weiss R., 2022, USA, English [24]	N	Y	Y	N	Y	NA	Y	Y
Melville et al., 2019, USA, English [25]	N	Y	Y	Y	Y	NA	Y	Y

N: no, Y: yes, NA: not applicable, The timing for the outcomes to occur is considered for short-term outcomes.

### 2.9. Data Analysis

Individual patient data were aggregated, and descriptive statistics were performed (MS Excel 2016).

## 3. Results

**Figure 1:**
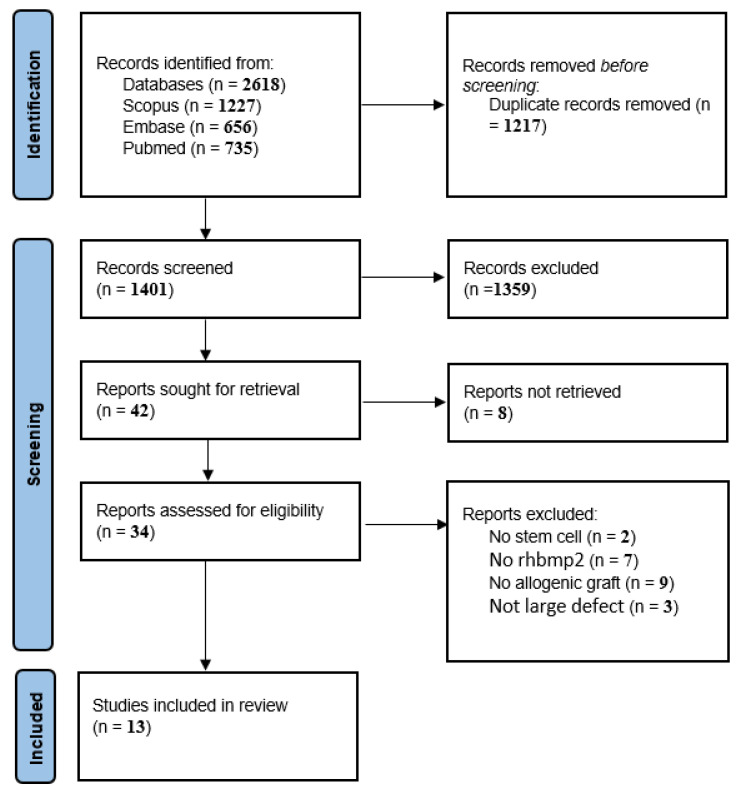
The PRISMA flowchart of included studies.

Figure 1 illustrates the PRISMA flow diagram for the study selection process at each level [10]. In the initial search, 2618 papers were obtained through PubMed, Scopus, and Embase. After duplication removal, 1401 papers remained the titles and abstracts of which were assessed for eligibility. A total of 1359 papers were removed by reading the title and abstract. Full texts were retrieved for the remaining 34 papers. Of those, 21 papers were excluded with reason. Finally, 11 papers were found to be eligible to be included in the current study.

### Study Characteristics

The characteristics of the included materials are shown in Table 4. Regarding the study type, eight case reports, three case series, and two clinical trials were included. A total of 144 cases were enrolled in this systematic review. The mean age of the patients was 43.34 (age range: 9–89). All incoming articles reported age. Seventy-one cases were male, and seventy-three were female. The mean follow-up time was 24.2 months with a range of 6 to 60 months. The included materials were published between the years 2008 and 2022 in the following countries: USA, India, Oman, Turkey, Israel, and Saudi Arabia.

Among the articles reviewed, two methods of immediate surgery and two-stage surgery were performed.

In 2016, a retrospective case study by Melville et al. [14] treated five patients with large mandibular defects caused by tumor ablation. The average amount of mandible defects was between 3.5 and 8 cm, which was treated with a combined method of freeze-dried cortical-cancellous bone and rhBMP-2 and BMAC in one session at the same time as removing the tumor with an intra-oral approach. With the same method of treatment, Melville et al. reported the treatment of a large maxillary defect that had been damaged due to trauma. This was a novel technique for large maxillary defects combining conventional techniques and tissue engineering techniques to create a custom-made graft utilizing in situ tissue engineering [16]. Schlund and colleagues reported a similar technique in a 33-year-old patient; they vascularized the allogenic graft with a radial forearm free flap to overcome poor vascularization in tissue-engineered allogeneic bone [19]. 

N. Ali et al. [22] reported a success rate of 88% by treating 24 surgical cases with a combination of allogeneic transplantation and Melville-like proteins and stem cells.

J. Johnson et al. [21] and her colleagues combined costochondral rib graft, allogeneic bone, BMAC, and recombinant human morphogenetic protein-2 in an 11-year-old patient with a 3.4 × 4.2 × 3.1 cm defect. A 100% success has been reported in a 1-year study.

Using the abovementioned combination, Kamal et al. [20] used titanium mesh for better bond results. Melville et al. [18] in another retrospective study treated 34 cases of tumor-like ameloblastoma, ossifying fibroma, odontogenic keratocyst (OKC), etc., using a non-resorbable titanium mesh or resorbable poly(L-lactide) (PLLA) or poly(D, L-lactide) (PDLLA) membrane addition of the mentioned combination bond, it was found that the graft is exceptionally vulnerable to bacterial contamination and also any patients with a history of uncontrolled health disease, chemotherapy, or radiation therapy negatively affect the graft’s viability.

RE Marx [27,28] and colleagues in two separate studies with two techniques and changing the amount of stem cells and their type compared the results to autogenous transplantation. In these two studies, the success rate was 97.4% to 40% compared to autogenous transplantation. The noticeable complication with this technique was edema, which was graded as nearly twice that of the autogenous graft and lasted nearly twice as long. They also stated that, in a series of cases, there is still a need to synchronize this technique with the autogenic technique and significantly more swelling.

## 4. Discussion

Alveolar defects caused by oncologic resection or trauma often involve extensive volumetric bone loss in the vertical and horizontal dimensions. If remaining untreated, they can lead to noticeable quality-of-life, nutritional, and speech issues [31,32]. The reconstruction of these hard tissue defects for shaping the appropriate facial form and functional rehabilitation poses a significant challenge for oral and maxillofacial surgeons. Successful reconstruction with a reasonably high long-term success rate (up to 70%) has been achieved with autogenous bone grafts. For decades, autografts as a natural biomaterial have been considered the gold standard due to superior osteoinductivity, osteoconductivity, and osteogenesis, compared with other types of materials [20,33]. Osteo-cutaneous free flaps, especially fibula free flaps, are the most common autografts used for crucial-sized grafts [19].

They also have shown histocompatibility and avoidance of immune rejection [34]. Morbidities in graft harvesting sites and bone transplantation sites, increased surgical time, and prolonged hospitalization are the main drawbacks of the conventional technique. Extensive graft harvesting from extra-oral sites has an increased risk of hematoma, pain and sensory disturbances, herniation of abdominal content, pelvic instability, and infections. Likewise, in huge defects, longer than 6 cm, the increased failure rate is not out of the question [35,36]. 

A promising alternative method to address a wide range of maxillofacial scenarios is tissue engineering. The strategies used in tissue engineering based on the use of cells, scaffolds, and bioactive molecules encompass tissue and organ regeneration [37]. Tissue engineering for maxillofacial bone defects is most successful in osteogenesis when mimicking both the macro- and micro-environment. Current literature supports the use of a biomimetic, bioactive osseointegrative customized scaffold according to the defect accompanied by growth factors and stem cells [38]. In recent years, a new tissue engineering technique using a combination of allogenic bone, BMAC, and rhBMP-2 has been introduced and advocated by Melville et al. for immediate reconstruction of large maxillofacial defects with less invasiveness, less intraoperative time, lower cost, and minimum/no donor site morbidities than conventional autografting methods [15].

The three basics of successful regeneration in this technique are allogeneic bone (scaffolding), BMAC (stem cells), and rhBMP-2 and platelet-rich plasma/platelet-rich fibrin (cell signaling for the promotion of stem cell migration and differentiation into osteoblasts). 

The scaffold is a three-dimensional framework on which cells can adhere and proliferate. A good scaffold to reconstruct bone is bone; allografts such as humeral bone have the desired strength to bear the mastication loads [19,39].

BMAC is a rich source of MSCs and osteoprogenitor cells, cytokines, and growth factors that can be derived from the tibia or iliac crests and delivered to bony defects [27]. BMAC is an affordable, easy-to-harvest, and safe technique to collect a considerable number of mesenchymal stem cells, and the results with this technique are comparable with an autograft alone [40,41].

rhBMP-2 added to allografts was used for alveolar reconstruction defects and sinus floor augmentation successfully, but recent experiments also suggest several clinical benefits of the off-label use of rhBMP-2 in the reconstruction of critical-sizedmaxillofacial defects. 

The included materials reported both immediate and delayed reconstruction with a transoral or extraoral approach. Although the preferred technique was mostly an immediate reconstruction, in cases with potential extensive soft tissue loss after surgery, delayed reconstruction was conducted. Sufficient soft tissue is a crucial factor for success rate; the required soft tissue volume would allow for a primary watertight tension-free closure to prevent bacterial contamination. If the amount of soft tissue seemed insufficient, a vascularized free flap was performed and later followed by delayed tissue-engineered reconstruction. Special care must be taken with an intraoral approach as tissue-engineered bone grafts are highly vulnerable to salivary leakage and bacterial contamination of the graft [18]. 

Literature on the use of rhBMP-2 in large maxillofacial defects in children is scarce. Only three papers included children (9–18 years) in their studies [17,18,21]. Although the predictability and safety of the combination of allogenic bone, BMAC, and BMP for reconstruction after resection of benign tumors in adult patients have been demonstrated, their use in children is still in dispute. The US Food and Drug Administration has warned about the use of BMP in patients with developing skeletons [42]. Use should be judicious as complications and long-term outcomes cannot be validated until larger studies on pediatrics be conducted.

Recently, a similar study has been conducted with an aim of reconstruction of critical-size tibia defects in a sheep model. The bone substitute in combination with endothelial progenitor cells (EPC), mesenchymal stem cells (MSC), and with (or without) growth factors BMP-2 was prevascularized and transplanted into a critical-size bone defect in 17 sheep models. They used an AV loop as an even less invasive approach, compared to a forearm free flap, for axial vascularization. During the first and third months after transplantation at the defect, good success was achieved [43].

There has been some evidence for the potential of immediate tissue engineering techniques to be an alternative treatment for the current gold standards, transplantation of vascularized autologous bone harvested from unharmed areas, in routine practice.

### Limitations of the Technique and Future Implications

Despite the growing body of literature and advances in the reconstruction of critical-sized maxillofacial defects, the technique has a number of shortcomings and several challenges ahead. 

One negative point of added rhBMP-2 is inevitable post-surgical swelling and edema attributed to the inflammatory cytokine-like nature of rhBMP-2, on which steroids have little impact. Therefore, pre-surgical precautions are warranted. rhBMP-2, when combined with absorbable collagen sponges as a carrier, provides a continuous release of the protein into the bone formation environment for three weeks after the surgery. The complication is blocked vascular growth and soft tissue compression at the bone regeneration site [44].

Nevertheless, some contraindications are listed for BMAC harvestings, such as cases with congenital disorders, metabolic diseases, malignancy, or a history of trauma in the harvesting site. Caution should be exercised in young patients (<18 years) [24]. Non-vascularized allografts have a high vulnerability to bacterial contamination [19]. 

Inadequate defect fit of scaffolds and personalized, customized substitute devices is another challenge to be faced, as the process may require multiple steps/device parts [15]. In addition, current techniques only allow for homogeneous bony structures regeneration, while clinical scenarios in craniofacial defects caused by tumor ablation or trauma mostly require the engineering of multiple tissues, which include soft, hard, and nerve tissue.

The temporomandibular joint as an osteochondral unit containing bone, cartilage, and transitional layers is one example. In complex cases of mandibular or zygomatic arch defects, reconstruction of the temporomandibular joint (TMJ) may be required. The TMJ has poor regenerative capacity due to the avascular nature of cartilage. 

The emergence of three-dimensional printing (3DP) technologies made notable progress in the regeneration of complex heterogeneous defects. This technology enables individualized substitute device construction [25]. The multilayer scaffold design creates a vascular network for better oxygen diffusion and waste exchange in heterogeneous defects [31].

The current literature is encouraging but as yet is too scarce to allow a firm conclusion to be drawn. With the knowledge of the possibility afforded for the future reconstruction of large maxillofacial defects, further studies with large sample sizes and long-term follow-ups are warranted to validate the routine use of this technology in the maxillofacial field.

## 5. Conclusions

The reviewed technique combines the allogenic graft as a biologic scaffold with bone marrow aspirate and rhBMP-2 to create a custom-made graft. 

The current literature demonstrates the potential for using large-scale transplantable, vascularized, and customizable bone with the aim of reconstructing large maxillofacial bony defects in short-term follow-ups. This approach might be an alternative to the current therapeutic clinical options that include vast autogenous bone harvest and many patient morbidities, although, further clinical trials with larger sample sizes in long-term follow-ups are needed to draw a firm conclusion.

## Figures and Tables

**Table 1:** Search strategy.

PubMed		
1	(“Bioengineering” [Mesh]) OR (“Bioengineering material” [Title/Abstract]) OR (“osteogenic scaffold” [Title/Abstract]) OR (“tissue engineering” [Title/Abstract]) OR (“Tissue Engineering” [Mesh]) OR (“Bone Morphogenetic Proteins” [Mesh]) OR (“Bone Morphogenetic Proteins” [Title/Abstract]) OR (“Mesenchymal Stem Cells” [Mesh]) OR (“Bone Mesenchymal Stem Cells” [Title/Abstract]) OR (“beta-tri calcium phosphate” [Title/Abstract]) OR (“Bone Morphogenetic Protein 2” [Mesh]) OR (rhBPM2) OR (rhBPM-2)	149,201
2	(Mandible[Title/Abstract]) OR (Mandibular[Title/Abstract]) OR (Maxilla[Title/Abstract]) OR (Maxillary[Title/Abstract]) OR (Maxillofacial[Title/Abstract])	175,429
3	(“Reconstructive Surgical Procedures” [Mesh]) OR (Reconstruct[Title/Abstract]) OR (Augment[Title/Abstract])	558,576
1 AND 2 AND 3	(TITLE-ABS-KEY (bioengineering) OR TITLE-ABS-KEY (“osteogenic scaffold”) OR TITLE-ABS-KEY (“osteogenic scaffolds”) OR TITLE-ABS-KEY (“tissue engineering”) OR TITLE-ABS-KEY (“Bone Morphogenetic Proteins”) OR TITLE-ABS-KEY (“Bone Morphogenetic Protein”) OR TITLE-ABS-KEY (“Mesenchymal Stem Cells”) OR TITLE-ABS-KEY (“Bone Mesenchymal Stem Cells”) OR TITLE-ABS-KEY (“beta-tri calcium phosphate”) OR TITLE-ABS-KEY (“Bone Morphogenetic Protein 2”) OR TITLE-ABS-KEY (rhbpm2) OR TITLE-ABS-KEY (rhbpm-2)) AND (TITLE-ABS-KEY (mandible) OR TITLE-ABS-KEY (mandibular) OR TITLE-ABS-KEY (maxilla) OR TITLE-ABS-KEY (maxilla) OR TITLE-ABS-KEY (maxillary) OR TITLE-ABS-KEY (maxillofacial)) AND (TITLE-ABS-KEY (“Reconstructive Surgical Procedures”) OR TITLE-ABS-KEY (reconstruct) OR TITLE-ABS-KEY (augment) OR TITLE-ABS-KEY (reconstruction) OR TITLE-ABS-KEY (augmentation))	735
Scopus		
1	TITLE-ABS-KEY (bioengineering) OR TITLE-ABS-KEY (“osteogenic scaffold”) OR TITLE-ABS-KEY (“osteogenic scaffolds”) OR TITLE-ABS-KEY (“tissue engineering”) OR TITLE-ABS-KEY (“Bone Morphogenetic Proteins”) OR TITLE-ABS-KEY (“Bone Morphogenetic Protein”) OR TITLE-ABS-KEY (“Mesenchymal Stem Cells”) OR TITLE-ABS-KEY (“Bone Mesenchymal Stem Cells”) OR TITLE-ABS-KEY (“beta-tri calcium phosphate”) OR TITLE-ABS-KEY (“Bone Morphogenetic Protein 2”) OR TITLE-ABS-KEY (rhbpm2) OR TITLE-ABS-KEY (rhbpm-2)	273,893
2	TITLE-ABS-KEY (mandible) OR TITLE-ABS-KEY (mandibular) OR TITLE-ABS-KEY (maxilla) OR TITLE-ABS-KEY (maxillary) OR TITLE-ABS-KEY (maxillofacial)	275,089
3	TITLE-ABS-KEY (“Reconstructive Surgical Procedures”) OR TITLE-ABS-KEY (reconstruct) OR TITLE-ABS-KEY (augment) OR TITLE-ABS-KEY (reconstruction) OR TITLE-ABS-KEY (augmentation)	1,114,947
1 AND 2 AND 3	(TITLE-ABS-KEY (bioengineering) OR TITLE-ABS-KEY (“osteogenic scaffold”) OR TITLE-ABS-KEY (“osteogenic scaffolds”) OR TITLE-ABS-KEY (“tissue engineering”) OR TITLE-ABS-KEY (“Bone Morphogenetic Proteins”) OR TITLE-ABS-KEY (“Bone Morphogenetic Protein”) OR TITLE-ABS-KEY (“Mesenchymal Stem Cells”) OR TITLE-ABS-KEY (“Bone Mesenchymal Stem Cells”) OR TITLE-ABS-KEY (“beta-tri calcium phosphate”) OR TITLE-ABS-KEY (“Bone Morphogenetic Protein 2”) OR TITLE-ABS-KEY (rhbpm2) OR TITLE-ABS-KEY (rhbpm-2)) AND (TITLE-ABS-KEY (mandible) OR TITLE-ABS-KEY (mandibular) OR TITLE-ABS-KEY (maxilla) OR TITLE-ABS-KEY (maxilla) OR TITLE-ABS-KEY (maxillary) OR TITLE-ABS-KEY (maxillofacial)) AND (TITLE-ABS-KEY (“Reconstructive Surgical Procedures”) OR TITLE-ABS-KEY (reconstruct) OR TITLE-ABS-KEY (augment) OR TITLE-ABS-KEY (reconstruction) OR TITLE-ABS-KEY (augmentation))	1227
Embase		
1	bioengineering:ti,ab,kw OR ‘osteogenic scaffold’:ti,ab,kw OR ‘tissue engineering’:ti,ab,kw OR ‘bone morphogenetic protein’:ti,ab,kw OR ‘mesenchymal stem cell’:ti,ab,kw OR ‘beta-tri calcium phosphate’:ti,ab,kw OR ‘bone morphogenetic protein 2′:ti,ab,kw OR rhbpm2:ti,ab,kw	115,437
2	mandible:ti,ab,kw OR ‘jaw disease’:ti,ab,kw OR mandibular:ti,ab,kw OR maxilla:ti,ab,kw OR maxillary:ti,ab,kw OR ‘maxillofacial disorder’:ti,ab,kw OR maxillofacial:ti,ab,kw	202,168
3	‘reconstructive surgery’:ti,ab,kw OR reconstruct:ti,ab,kw OR reconstruction:ti,ab,kw OR augment:ti,ab,kw OR augmentation:ti,ab,kw	473,086
1 AND 2 AND 3		656

**Table 2:** Excluded articles with reasons.

Articles (First Author, Year, Title)	Reason for Exclusion
N.M.A. Lopes, 2012, Use of rhBMP-2 to reconstruct a severely atrophic mandible: A modified approach	tricalcium phosphate instead of allogenic bone
Schuckert KH, 2009, Mandibular Defect Reconstruction Using Three-Dimensional Polycaprolactone Scaffold in Combination with Platelet-Rich Plasma and Recombinant Human Bone Morphogenetic Protein-2: De Novo Synthesis of Bone in a Single Case	de novo not allogenic
Jörg Wiltfang, 2016, man as a Living Bioreactor: Prefabrication of a Custom Vascularized Bone Graft in the Gastrocolic Omentum	bovine bone not allograft
Rômulo Maciel Lustosa, 2014, Mandible reconstruction using rhBMP-2: case report and literature review	bovine bone xenograft not allograft
G. K. Sándor,2013, Adipose stem cell tissue-engineered construct used to treat large anterior mandibular defect: a case report and review of the clinical application of good manufacturing practice-level adipose stem cells for bone regeneration (β-tricalcium phosphate)	(β-TCP) granules not allogenic
K. Mesimäki, 2009, Novel maxillary reconstruction with ectopic bone formation by GMP adipose stem cells	beta-tricalcium phosphate not allogenic
B. Zamiri, 2013, Reconstruction of human mandibular continuity defects with allogenic scaffold and autologous marrow mesenchymal stem cells	ex-vivo MSC
L. M. S. Zanettini, 2018, use of Recombinant Human Bone Morphogenetic Protein-2 Associated With Lyophilized Bovine Bone in Reconstruction of Atrophic Maxilla	bovine not allograft
M. Albanese, 2012, Fresh-frozen human bone graft to repair defect after mandibular giant follicular cyst removal: A case report. Cell and Tissue Banking.	no rhBMP
R. Bertolai, 2015, Bone graft and mesenchimal stem cells: Clinical observations and histological analysis. Clinical Cases in Mineral and Bone Metabolism.	mesenchymal stem cells engineered freeze-dried bone allografts/no rhBMP
C. M. Clokie, 2008, Reconstruction of 10 major mandibular defects using bioimplants containing BMP-7	no rhBMP
M. Cicciù, 2012,Protein-Signaled Guided Bone Regeneration Using Titanium Mesh and Rh-BMP2 in Oral Surgery: A Case Report Involving Left Mandibular Reconstruction after Tumor Resection	no allograft
S. C. Desai, 2013, Use of Recombinant Human Bone Morphogenetic Protein 2 for Mandible Reconstruction	no BMA
C. M. Misch, 2015, Vertical Bone Augmentation Using Recombinant Bone Morphogenetic Protein, Mineralized Bone Allograft, and Titanium Mesh: A Retrospective Cone Beam Computed Tomography Study	not large defect
B. B. Kim, 2014, Hybrid mandibular reconstruction technique: Preliminary case series of prosthetically-driven vascularized fibula free flap combined with tissue engineering and virtual surgical planning	no rhBMP2
Mark C. Fagan, 2008, Simultaneous hard and soft tissue augmentation for implants in the esthetic zone: Report of 37 consecutive cases.	no rhBMP2
B. Haj Yahya, 2020, Non-Autogenous Innovative Reconstruction Method Following Mandibulectomy	defect size is small
H. I. Canter, 2007, Reconstruction of mandibular defects using autografts combined with demineralized bone matrix and cancellous allograft	bone harvest included
C. Loperfido, 2014, Severe mandibular atrophy treated with a subperiosteal implant and simultaneous graft with rhBMP-2 and mineralized allograft: a case report	no stem cell
A. Deshmukh, 2015, Bilateral maxillary sinus floor augmentation with tissue-engineered autologous osteoblasts and demineralized freeze-dried bone [2]	defect size is small
M. S. Block, 2010, Use of Living Cell Construct to Enhance Bone Reconstruction: Preliminary Results	no rhBMP

**Table 4:** Characteristics of included materials.

First Author/Year/Country of Origin/Language	Type of Study	Number of Cases/Duration of Follow Up	Mean Age/Sex	Summary of Method	cause of Defect/Size of Defect/Filling Rate of Defect (Bone Gain)	Mesenchymal Stem Cells Harvesting Site	Success Rate
James C. Melville, 2016, Houston, English [15]	Case report	5 cases/12–14 months	18–66 years old/3 men and 2 women	the freeze-dried cortical-cancellous bone in combination with large rhBMP-2 (12 mg)/absorbable collagen sponge (ACS) and 120 mL of BMAC obtained from anterior or posterior hip were mixed homogeneously. 10 mL of crushed cortical cancellous bone for each 1 cm length of the defect.	benign mandibular tumors with no history of chemotherapy or radiation to the mandible (Ossifying fibroma, Desmoplastic, Juvenile ossifying fibroma)/3.5 to 8.0 cm/10–14.5 mm and regenerated bone height was in the range of 22–26 mm	BMAC was harvested from either the bilateral anterior iliac crest or unilateral posterior iliac crest	100% success
James C. Melville, 2017, Houston, English [16]	Case report	1 case/6 months	45 years old/woman	radial forearm fasciocutaneous flap combined with a tissue-engineered bone graft consisting of allogeneic bone, rhBMP-2, and BMAC.	trauma (deficient projection of the left malar region, loss of left maxillary ridge alveolar bone, loss of dentition and upper eyelid ptosis, and lower eyelid ectropion)	BMAC form the iliac crest	100% success
Robert E Marx, 2014, USA, English [26,27]	Case report	40 case/6 months	mean age 57 years (19–78 years)/22 men and 12 women	in situ tissue-engineered graft containing 54 ± 38 CD34+ cells/mL along with 54 ± 38 CD44+, CD90+, and CD105+ cells/mL together with rhBMP-2 in an absorbable collagen sponge (1 mg/cm of defect) and crushed cancellous allogeneic bone.	/6- to 8-cm continuity defects/trabecular bone area of 36 ± 10%, versus 67 ± 13% for group B	four puncture sites in the bilateral anterior iliac crest	Group A: 40% success rate Group B: 100% success rate
J. C. Melville, 2014, Houston, English [17]	retrospective study	9 cases/4 years	mean age 23.7-year-old (3 patients under the age of 17)/5 men, 4 women	freeze-dried cortical cancellous bone was obtained used in combination with 12 mg of rhBMP-2/ACS and 120 cc of Bone Marrow Aspirate Concentrate	ameloblastoma, OKC, Myxoma, Ossifying Fibroma and Central Giant Cell Tumor/4 cm to 12 cm	anterior hip	100% success rate
Robert E Marx, 2013, USA, English [28]	clinical trial	20 cases/6 months	Mean age 58 and 62/5 men, 15 women	two types of grafts in large vertical maxillary defects: a composite graft of recombinant human bone morphogenetic protein-2/acellular collagen sponge (rhBMP-2/ACS), crushed cancellous freeze-dried allogeneic bone (CCFDAB), and platelet-rich plasma (PRP); and size-matched 100% autogenous grafts	horizontal defects/1 cm vertical deficiency and 1 cm horizontal deficiency spanning at least a four-tooth length/2-mm-diameter bone core (bone area 59 ± 12% and 54 ± 10%)	tibia plateau or anterior ilium	composite graft: 97.4% success rate autogenous grafts: 100% success rate
James C. Melville, 2019, Houston, English [18]	retrospective case	34 cases/5 years	mean age 37.79 ± 20.4 (9–89 years old)/19 men, 15 women	first, BMAC was obtained from the patient’s anterior or posterior hip using the Harvest Bone Marrow Aspirate Concentrate System. Second, a medium to large rhBMP-2 kit was used, according to size of the defect (a large kit was used for defects greater than 6 cm). Third, corticocancellous bone (MTF Biologics. Edison, NJ, USA) was milled down to a 1.0- to 2.0-mm particulate graft. Finally, a non-resorbable titanium mesh or resorbable poly (L-lactide) (PLLA) or poly (D, L-lactide) (PDLLA) membrane was used as the containment system for the graft.	ablative tumor surgery or traumatic accidents (ameloblastoma, ossifying fibroma, odontogenic keratocyst (OKC), and sclerosing osteomyelitis, odontogenic myxoma, giant cell tumor associated with hyperparathyroidism, and central giant cell granuloma)/continuity defect 5.61 ± 2.92 and Noncontinuity defect 4.77 ± 3.33/mean height 2.12 ± 0.44 Mean width 1.53 ± 0.55	anterior or posterior hip	Continuity defect 90% Noncontinuity defect 100%
Matthias Schlund, 2019, Oman, English [19]	clinical report	1 cases/1 years	33-year-old/men	using a fresh-frozen humeral allograft as scaffold seeded with progenitor cells collected through iliac bone marrow aspirate and vascularized with a radial forearm free flap	severe craniofacial trauma resulting in several fractures of the facial skeleton including a comminuted mandibular fracture from left parasymphysis to left angle	iliac bone marrow aspirate	100% success rate
Kamel Alraei, 2020, Saudi Arabia, English [20]	case report	1 case/4 years	27 years old/female	reconstruction of the mandibular defect using rhBMP-2 combined with bone marrow aspirate concentrate (BMAC), and an allograft with a titanium mesh. placed a total of 12 mg of rhBMP-2 in four absorbable collagen sponges	a calcifying, cystic, odontogenic tumor (a Pindborg tumor)/2.7 mm	60 cc of bone marrow from left iliac crest	Success rate 100%
Jeanette Johnson, 2016, Texas, English [21]	Case report	1 case/1 years	11 years old/female	reconstruction using a costochondral rib graft, allogeneic bone, bone marrow aspirate concentrate, and recombinant human morphogenetic protein-2.	unilocular radiolucent lesion of the posterior left mandible/3.4 × 4.2 × 3.1 cm	bone marrow aspirate concentrate	Success rate 100%
N. Ali, 2018, Texas, English [22]	Retrospective study	24 cases/3 years	Mean age 32.1 years old (11–65 years old)/ 14 men and 10 women	freeze-dried cancellous bone was obtained from MTF and used in combination with 12 mg of rhBMP-2/ACS, and 120 cc of BMAC was obtained from the patients	ablative tumor and trauma/ 5–17 cm/ 3.0 cm bone height and >1.0 cm bone width	anterior or posterior hip	88% success rate
Todd G. Carter, 2008, Seattle, English [23]	Case report	5 cases/22 months	42,43, 41, 81/2 men, 3 women	reconstruction with rhBMP-2. Because, in case 1, rhBMP-2 absorbed on a collagen sponge alone failed to regenerate bone, autogenous bone marrow, and allogenic bone were combined with rhBMP-2-impregnated collagen sponges to increase the osteogenic.	open and displaced left mandibular angle fracture, with an infection that required incision and drainage, multiple facial lacerations and a comminuted mandible fracture, osteomyelitis of the right mandible, radiolucent lesions in the mandible/4 cm, 4.5 cm	bone marrow from left iliac crest, bone marrow cells from the patient’s right anterior iliac crest.	-
Weiss R., 2022, USA, English [29]	Case report	2 cases/4 months	62-year-old woman/24-year-old woman	reconstruction with (1) corticocancellous bone chips (2) bone marrow aspirate, (3) rhBMP-2 A combined intraoral and extraoral approach was used to allow access. Cadaveric rib allograft was secured to the inferior aspect of the reconstruction plate. Nerve allografts were secured to the inferior alveolar nerve stumps and a water-tight closure	excisional biopsy of ameloblastoma 8 years earlier, segmental defect 6.0 × 5.0 × 3.7 cm/no past medical history, 4.4 × 2.1 × 2.1 cm	bone marrow was harvested via trochar and cannula from the anterior iliac crest	100%
Melville et al., 2019, USA, English [30]	Case report	1 case/6 months	45-year-old female	exposing and preparing the alveolar defect as well as harvesting the BMAC from the iliac crest and harvesting the radial forearm. crushed corticocancellous bone and rhBMP-2 were mixed with BMAC. The tissue-engineered graft was placed and packed onto the defect. The radial forearm was then sutured around and over the graft.	post-traumatic maxillary alveolar ridge defect/size not mentioned	bone marrow can be aspirated from the anterior ilium	100% success

## Data Availability

Not applicable.
